# Development of a Plasmonic Sensor for a Chemotherapeutic Agent Cabazitaxel

**DOI:** 10.1021/acsomega.2c05327

**Published:** 2022-12-26

**Authors:** Süleyman Aşır, Buse Uğur, Mitra Jalilzadeh, Ilgım Göktürk, Deniz Türkmen

**Affiliations:** † Department of Materials Science and Nanotechnology Engineering, Near East University, Mersin 10, Nicosia99138, North Cyprus, Turkey; ‡ Department of Biomedical Engineering, Near East University, Mersin 10, Nicosia99138, North Cyprus, Turkey; § Department of Chemistry, Faculty of Science, Hacettepe University, Beytepe, Ankara06800, Turkey

## Abstract

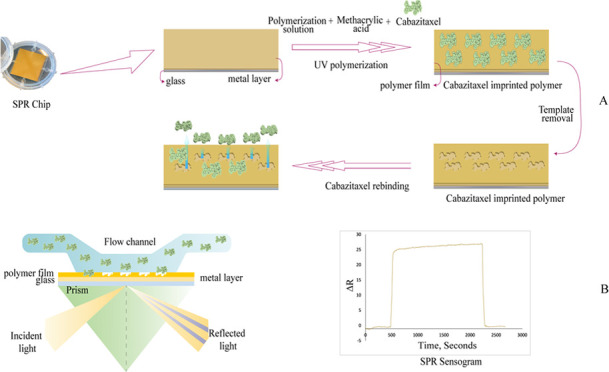

Drug dosage is a crucial subject in both human and animal treatment. Administering less drug dosage may prevent treatment or make it less effective, and high drug dosage may cause a heightened risk of adverse effects, or in some cases, cost a patient’s life. Also, even when the dosage is administered carefully, metabolic differences may cause different effects on different patients. Because of these considerations, monitoring drug dosage in the body is a critical and significant requirement in the health industry. Within the scope of this study, a reusable surface plasmon resonance (SPR) chip with fast response, high selectivity, and no pretreatment is produced for the chemotherapeutic agent cabazitaxel. A cabazitaxel-imprinted nanofilm was synthesized on the sensor chip surface and characterized by atomic force microscopy, ellipsometry, and contact angle measurements. Standard cabazitaxel solution and an artificial plasma sample were used for the kinetic analysis. Docetaxel, methylprednisolone, and dexamethasone were analyzed for their selectivity experiment. In addition, the repeatability and storage durability of the sensor were also evaluated. As a result of the adsorption studies, the limit of detection and limit of quantitation values were found to be 0.012 and 0.036 μg/mL, respectively. High-performance liquid chromatography analysis was used to validate the response of the cabazitaxel-imprinted sensor.

## Introduction

In the European Union (EU) countries, cancer is the leading cause of death among people younger than 65 years of age.^1^ Breast, colon, lung, and prostate cancers account for 50% of all cancer diagnoses. Prostate cancer (22.2% of all males, lung, 14.8%, colorectum, 13.2%, bladder, 7.3%) is the most prevalent among men.^1^

In Europe, prostate cancer ranked as the fourth most prevalent cancer in 2020. The introduction of PSA testing in the early to mid-1990s, which is mainly responsible for the increase in prostate cancer incidence rates,^2^ led to a rapid increase in the detection of prostate cancers in their early stages during the early to mid-1990s.

Several solid cancers, including the prostate, may be linked to chronic inflammation. Prostate carcinogenesis may be aided by oxidative stress and reactive oxygen species produced from inflammation. The discovery of a urinary microbiome indicates that the prostate may regularly be exposed to a wide range of microorganisms, which could contribute to an inflammatory microenvironment.^3^ However, it has not yet been determined which of the many infectious agents found in prostate tissue samples or prostate secretions is responsible for damaging or inducing inflammation in the prostate.

Castration-resistant prostate cancer (CRPC) accounts for the majority of deaths.^4^ Several treatment options for CRPC are currently in use, including immunotherapy, radiotherapy, chemotherapy, vaccine therapy, and experimental therapies.^5−7^ Ongoing clinical trials have shown that chemotherapy is an effective treatment option. Docetaxel (DTX), cabazitaxel (CTX), estramustine, and mitoxantrone are examples of common chemotherapeutic agents.^8^ CTX inhibits tumor cell mitosis and prevents androgen receptor translocation into the nucleus. Recent evaluations have compared the reduced dose of CTX to the currently approved dose (25 mg/m^2^ body surface area).^8^

Some chemotherapeutic agents can cause severe neurological adverse effects, lowering the standard of living and limiting the amount that can be administered. Human tumors that are relatively resistant to chemotherapy and patients with advanced prostate cancer despite DTX treatment have shown promising results with the next-generation CTX.^9^ For example, CTX can cross the blood–brain barrier, whereas DTX and paclitaxel^10^ have low absorption potentials. Intriguingly, the neurotoxic effect of CTX was found to be lower than that of other taxanes such as DTX and paclitaxel.

The amount of medicine or drug administered in the body is determined with toxicology screening tests, where the life quality of patients was demonstrated to be severely affected by those toxicities.^11−13^ Such an analysis is done with instrumental methods for drugs/medicines.^14^ Using these methods in the analysis takes a long time, and it is laborious, which is not a wanted feature because late analysis means late diagnosis and late treatment, which may result in the patient’s death or serious harm to the patient. Various sensors have been developed in the last decade to address this issue.^15^ Sensors are small devices that can sense their environment and use them for chemical analysis.^16,17^ Their flexible structure and small size make them an alternative to classic laboratory analysis equipment. Also, making them reusable or disposable is possible, which is the desired feature in some cases.

Optical sensors can detect many biological and chemical substances directly, label-free, and in real time, having significant advantages over traditional analytical methods. Their advantages include accuracy, ease of use, low cost, high specificity, and sensitivity.^18,19^ Surface plasmon resonance (SPR) is one of the most prevalent optical sensor subclasses. Electrons in the conduction band oscillate collectively in resonance with the oscillating electric field of the incident light, causing SPR to occur.^20^ SPR’s lack of a label makes it ideal for studying molecular interactions rapidly, precisely, and sensitively.^21^ SPR sensors have a wide range of applications, including diagnosis, disease surveillance, enzyme-linked immunosorbent assay, diagnostic and therapeutic analysis, biomedical and processing industry, animal health, environmental control of pollution, and agriculture applications.^22−24^ Applying surface functionalization to a sensor can enhance its specificity against target molecules. In the molecular imprinting technique, macromolecules with target molecule-specific recognition sites can be synthesized.^25,26^ Molecularly imprinted polymers (MIPs) have numerous excellent characteristics, including stable chemical, physical, and mechanical properties, high pressure and temperature resistance, strong resistance to acids and alkalies, simple production, long-lasting performance, reuse, and recycling.^27,28^

**Figure 1:**
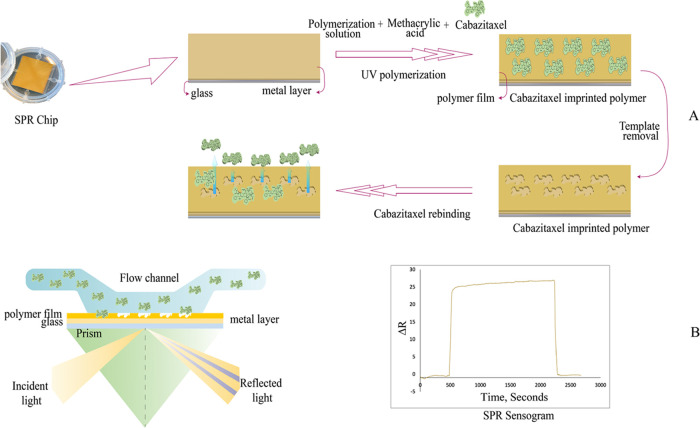
(A) Molecular imprinting of the template CTX on the surface of the SPR chip. (B) Working principle of an SPR-based sensor.

SPR-based sensors are used to simultaneously directly measure interactions between biomolecules without any marking,^29^ and the scheme of SPR-based sensor is demonstrated in Figure 1A. This technology is based on detecting minute changes in the refractive index of thin metal (Au, Ag) films brought about by the interaction of target compounds with a specific transducer via a change in the resonance angle. As shown in Figure 1B, the molecular imprinting technique is predicated on creating template-specific cavities in a cross-linked polymer matrix. These cavities can identify the size and shape of the target molecule. The removal of the template molecule by any desorption method reveals functional monomer groups at the correct positions, and a structure binding site to the target molecule is formed.

Here, CTX-imprinted methacrylic acid–ethylene glycol dimethylacrylate–hydroxyethyl methacrylate (CTX MIP) sensor was synthesized on the SPR chip surface as a synthetic receptor for CTX. Kinetic studies were carried out after the preparation and characterization of the polymeric nanofilm. CTX samples prepared at different concentrations were applied to the SPR sensor system, the binding kinetic parameters were evaluated, and the sensing performance was measured. Additionally, the CTX-spiked artificial plasma sample was analyzed then the selectivity and reusability experiments were performed. High-performance liquid chromatography (HPLC) analysis was used to validate the response of the CTX MIP sensor.

## Materials and Methods

### Materials

The gold SPR chips were supplied by GWC Tech (Madison) (Product code: SPR-1000-050, chip gold thickness: 50 nm, chip dimensions: 1 mm × 18 mm × 18 mm, *n*_glass_: 1.72). Sigma-Aldrich provided CTX, docetaxel (DTX), methylprednisolone (MP), dexamethasone (DEX), functional monomer methacrylic acid (MAA), ethylene glycol dimethacrylate (EGDMA), 2-hydroxyethyl methacrylate (HEMA), azobisisobutyronitrile, sodium chloride, and 2-propene-1-thiol. Merck KGaA, Darmstadt, supplied all solvents. Ultrapure water (18.2 MΩ·cm) was used in all solution preparation and dilution processes throughout the experiments.

### Design of the CTX MIP Sensor

Before the preparation of the CTX-imprinted (CTX MIP) and nonimprinted (NIP) sensors, the surface of the chips was cleaned with an acidic piranha solution. They were rinsed with an aqueous ethanol solution and dried at room temperature. After cleaning the gold chip surface, 3 mL of 2.0 mM 2-propene-1-thiol solution was dropped dropwise to form allyl mercaptan groups. The prepared chip was incubated for 2 h, and unbound 2-propene-1-thiol was removed by washing with ethanol. The chip was then dried in a vacuum oven. The CTX MIP sensor was prepared in three stages. The initial step was to modify the sensor surface using allyl. Preparing the CTX–MAA precomplex was the second stage. Finally, the CTX MIP sensor was created under controlled environments by combining the precomplex and polymerization mixtures on the modified chip surface.

A UV spectrophotometer (Thermo Scientific Genesys 10S UV–vis) was used to analyze the different (1/0.5, 1/1, 1/2, and 1/3) concentration ratios of the CTX–MAA complex to determine the optimal stoichiometric ratio of the CTX and MAA precomplex. The most excellent absorbance value was reported in the CTX–MAA acid combination at a 1/3 ratio. As a result, the CTX: MAA ratio was set at 1/3.

A polymerization solution containing HEMA, EGDMA, CTX–MAA complex in a 1/3 ratio, and azobisisobutyronitrile as initiator was created to prepare a CTX MIP nanofilm on the SPR chip surface. The solution was then aliquoted and dropped on the allylated gold surface of the SPR chip. UV light was employed at 25 °C (100 W, 365 nm) for 60 min (Figure 1B). MAA was mixed into the polymerization system without CTX to produce the NIP sensor rather than the CTX–MAA complex. Finally, the CTX MIP SPR and NIP sensor were washed in an aqueous ethanol solution, dried in a vacuum oven, and stored in a desiccator. Furthermore, CTX was removed from CTX-imprinted nanofilm-coated chips using a 0.5 M NaOH solution. The NIP sensor was produced using the same procedure without the addition of the CTX template analyte molecule.

### Characterization Studies

Atomic force microscopy (AFM, Nanomagnetics Instruments, U.K.), ellipsometer (Nanofilm EP3, Germany), and contact angle (CA, KRUSS DSA100, Hamburg, Germany) measurements were used to characterize the CTX MIP and NIP sensors. To investigate the depth of the surface, an ambient AFM was used in the tapping mode. Three-dimensional images were obtained by scanning the surface of the plasmonic sensors with high resolution. While the scanning speed of the images was 1 μm/s, an image was obtained from an area of 1 × 1 μm^2^. In addition, the thickness of the polymeric layer on the gold surface of the SPR chip was measured using an auto-nulling imaging ellipsometer. Finally, the contact angles of CTX MIP and NIP sensors were measured. During the process, the sessile drop method obtained the contact angle values, and the wettability was measured by dropping water on the surfaces. After dropping water into three different regions, images were taken in each region, and contact angles were determined.^30^

### Kinetic Studies of the CTX MIP Sensor

Kinetic analyses of CTX MIP and NIP sensors were performed using SPR imager II (GWC, Madison) with a flow rate of 150 μL/min and an operating wavelength of 800 nm. CTX was detected from the aqueous solution and artificial plasma samples by the CTX MIP sensor. First, CTX detection studies in varied aqueous media at different pH values (5, 6, 7.4, 9) were evaluated to establish the effective pH for the detection of CTX in the 0.05–150 μg/mL range. The detection time was nearly 10 min. The CTX MIP sensor was equilibrated with 0.5 M phosphate buffer at pH 6.0. After CTX adsorption on the sensor surface, the 0.5 M NaOH solution achieved desorption. The fluctuations in resonance frequency were tracked in real time and reached a steady-state equilibrium in 15 min. The aqueous solutions of DTX, MP, and DEX were used to test the selectivity of the CTX MIP sensor. The reusability of the CTX MIP sensor was evaluated by cycling equilibration–adsorption–desorption four times with CTX solutions containing 20.0 μg/mL water.

### Confirmation Analysis of the CTX MIP Sensor

To test the reliability and validity of the constructed SPR sensor, the experiments were carried out using artificial plasma. A Dionex Ultimate HPLC system with a photodiode array detector and a C18 (100 mm 4.6 mm i.d., 5 μm particle size) column was kept at 25 °C to achieve chromatographic separation. By carefully weighing 25 mg of CTX into a 25 mL volumetric flask containing a mobile phase, a stock solution of CTX (1000 μg/mL) was produced. Working standard solutions were made daily from the mobile-phase-infused stock solution by filtering them through a 0.45 μm membrane filter before injection. One thousand milliliters of water was used to dissolve 6.8 g of potassium dihydrogen phosphate, and 10 M potassium hydroxide was used to bring the pH level down to 5.0. Isocratic elution was carried out using phosphate buffer and acetonitrile (50/50, v/v). The flowing rate was 1 mL/min, and the length of operation was 10 min. The sample was injected into the HPLC system at a volume of 20 μL and 230 nm wavelength.^31^

## Results and Discussion

### Characterization of the CTX MIP Sensor

**Figure 2:**
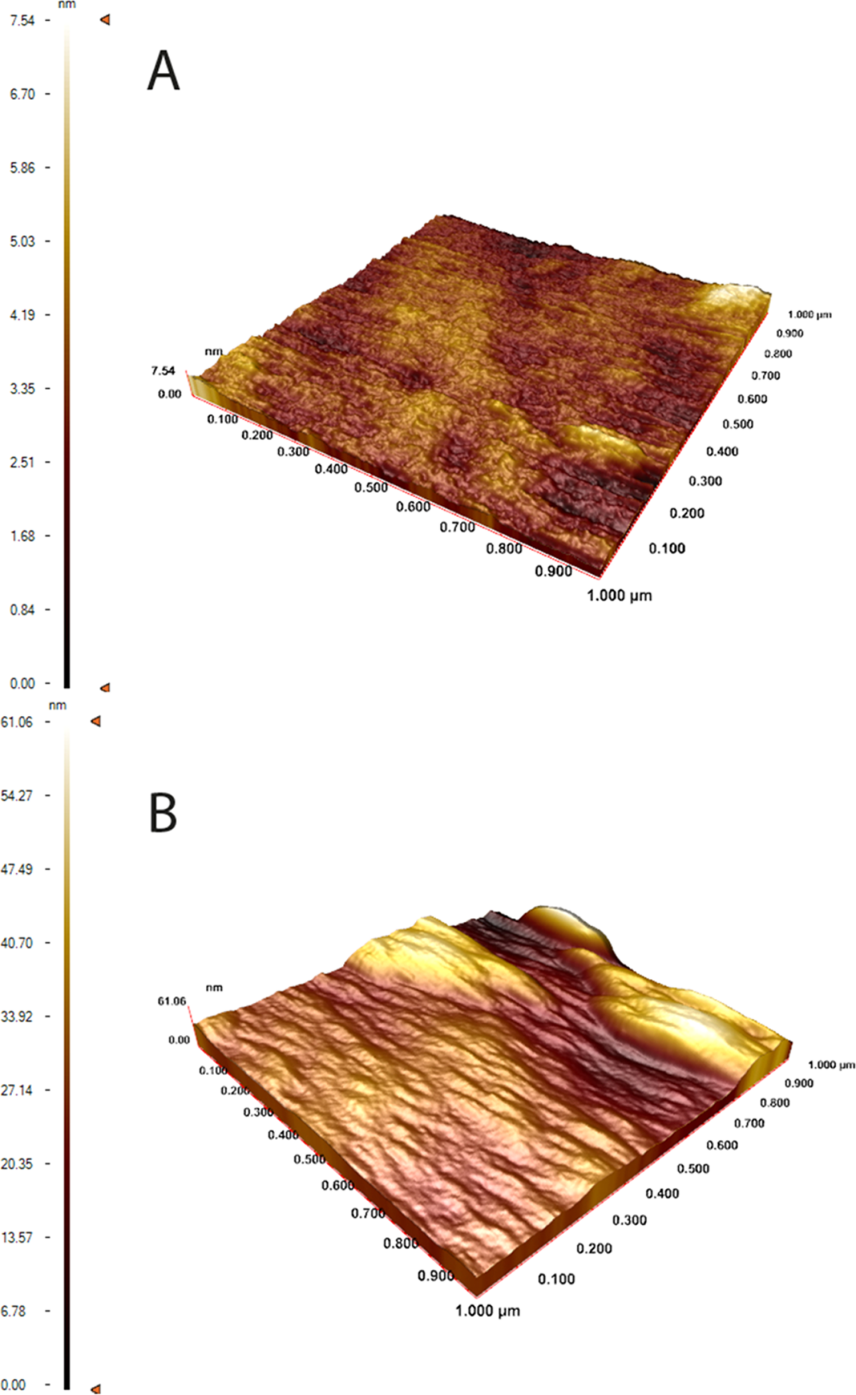
AFM images of (A) bare chip and (B) CTX MIP sensor surface.

AFM in a half-contact mode characterized the bare chip and CTX MIP sensor surface morphologies. Figure 2 shows AFM images of the bare surface and CTX MIP sensor. AFM images determined the surface depth of the bare chip to be 7.54 ± 2.17 nm, while the surface depth of the CTX MIP sensor was 61.06 ± 1.0 nm. The difference in surface depth values between the bare chip and the CTX MIP sensor indicates that the polymeric structure was successfully fabricated on the chip surface.

The thickness of the SPR sensor surfaces following each modification step was measured using ellipsometry. According to the results, the thickness of the unmodified and CTX MIP sensor surfaces was 8.9 ± 0.6 nm and 65.5 ± 0.9 nm, respectively. These outcomes matched those of the AFM. The findings of the AFM and ellipsometry measurements demonstrated the rough surfaces of the CTX MIP sensor surfaces, and the SPR sensor surfaces’ thickness differences demonstrated the imprinting process’s efficacy.

**Figure 3:**
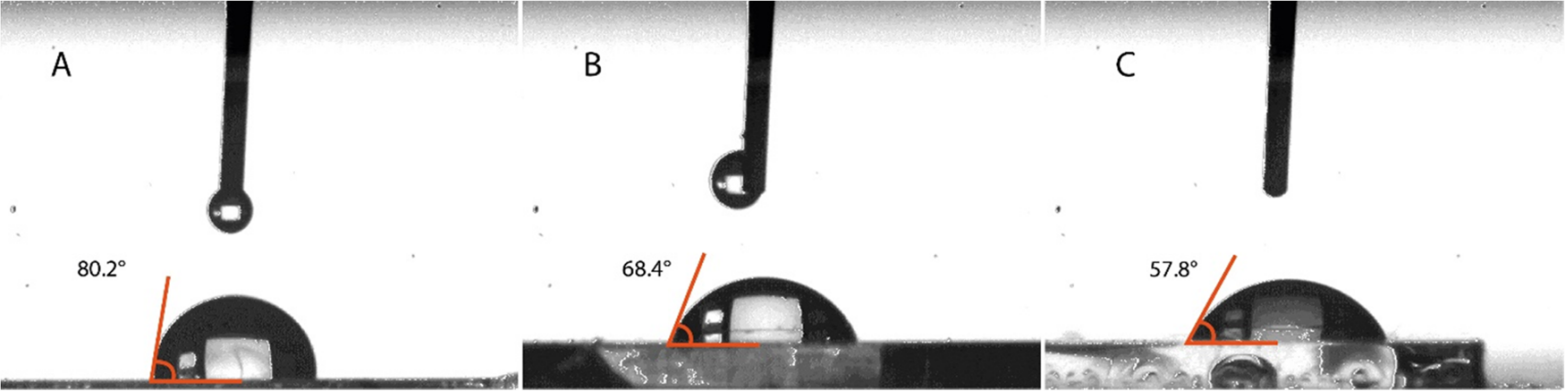
CA measurement of (A) bare chip, (B) ally-modified chip, and (C) CTX MIP sensor.

The CA images of the bare chip surface, the ally-modified chip surface, and the CTX MIP sensor surface are given in Figure 3. According to the results of the CA measurements, the CA value of the surface of the bare chip was recorded as 80.2 ± 1.5°, while the contact angle of the ally-modified chip was measured as 68.4 ± 2.3°. After CTX imprinting, the wettability of the CTX MIP sensor surface increases according to the contact angle value measured as 57.8 ± 1.2°.

### Aqueous Solution Study of the CTX MIP Sensor

#### Effect of pH and Imprinting Factor (IF)

The molecular imprinting method relies on the interaction of a functional monomer and a template to produce a complex, where a three-dimensional polymer network is created following the construction of this complex and the application of a cross-linking agent. When the template is removed from a polymer, it leaves behind specific recognition sites structurally, dimensionally, and functionally identical to the template molecule. Intermolecular interactions such as dipole–dipole interactions, hydrogen bonds, and ionic interactions between the template molecule and functional groups in the polymer matrix frequently drive the molecular recognition phenomenon. As a result, only the molecules from the template are recognized and bound by the resulting polymer. This recognition and binding strictly depend on the experimental conditions; one of the crucial ones is pH, which affects the structural properties of both the cavities and the template molecule.

**Figure 4:**
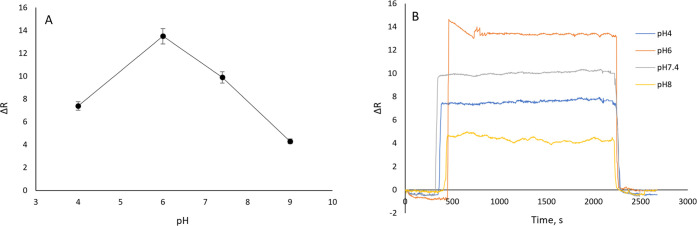
(A) Sensorgram responses with error bars. (B) Sensorgrams of CTX by designed CTX MIP sensor in different pH values (*C*: 20.0 μg/mL, *T*: 25.0 °C, repeated three times (*n* = 3)).

Figure 4 shows the impact of pH (4.0, 6.0, 7.4, and 9.0) on the adsorption of CTX to the polymer formed on the surface of the CTX MIP sensor. All tests and measurements were conducted three times, with the average data used for analysis (Figure 4A,B). The highest CTX adsorption took place at pH 6.0, as shown by the graph we obtained from the sensorgram. The interaction between CTX-imprinted polymers relied on hydrogen bonds. The MAA hydroxyl group (O–H) and CTX would not form hydrogen bonds because of the deprotonation impact of high pH, and the hydrogen bonding increases at low pH because of protonation; afterward, the hydrogen binding decreases at lower pH because of the saturation of binding sites.

As a result, the sensor’s selectivity was decreased, and ligand binding affinity to the template molecule varied with pH levels. The pH effects results’ relative standard deviation (RSD) was less than 1.29, showing repeatability.

#### Effect of CTX Concentration

**Figure 5:**
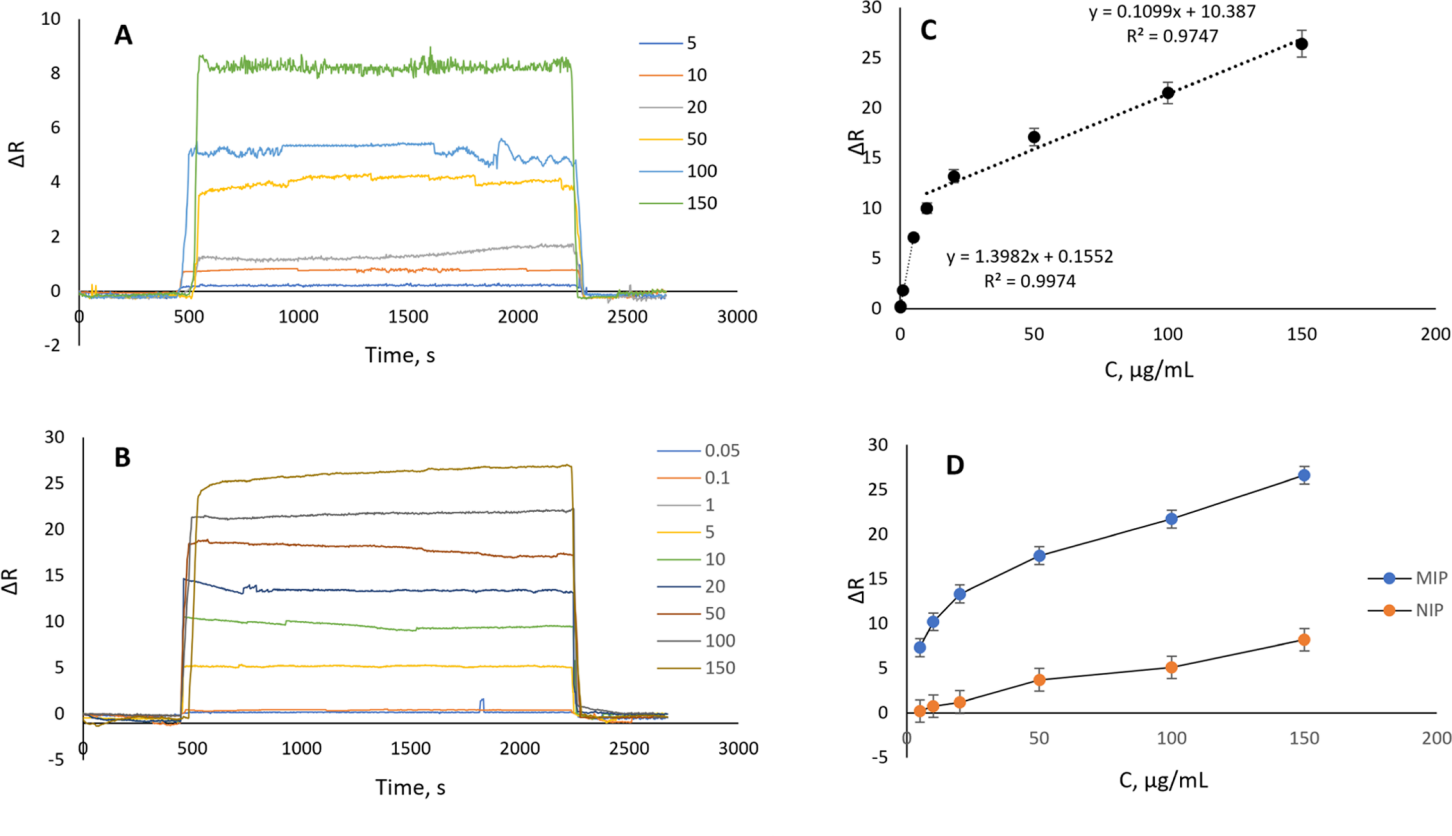
(A) NIP sensor response for CTX with a range of 5–150 μg/mL concentrations. (B) CTX MIP sensor response for CTX with a range of concentrations of 0.05–150 μg/mL. (C) CTX MIP sensor linear response for CTX with a range of concentrations of 0.05–5 μg/mL and 10–150 μg/mL. (D) Comparison of CTX MIP and NIP sensor response for CTX with a range of 5–150 μg/mL concentrations.

In Figure 5, we compared the NIP sensor and CTX MIP sensor with a range of CTX concentrations (5–150 μg/mL for the nonimprinted sensor and 0.05–150 μg/mL for the CTX-imprinted sensor) to assess the impact of imprinting on CTX adsorption and to estimate kinetic parameters. CTX-imprinted polymer-coated SPR nanosensor chips were used to conduct real-time CTX adsorption tests. The investigation was conducted with a CTX concentration range of 0.05–150.0 μg/mL. To equilibrate the sensors, we employed a phosphate buffer with a pH of 6.0. The SPR view program then computed the outcomes after delivering the solutions to the SPR sensor. It took 40 min to complete the adsorption, desorption, and regeneration procedures. Figure 5B,C shows the obtained sensorgrams and their calibration curves. The measurements for the limit of detection (LOD) and limit of quantification (LOQ) were calculated using the 3 and 10 s/m approaches. The results showed that the LOD and LOQ values were 0.012 and 0.036 μg/mL, respectively.

In the range of concentrations of 0.05–150 μg/mL, the correlation coefficients for the CTX MIP sensor are *y* = 1.3982*x* + 0.1552 with 99.7% accuracy in the concentration range of 0.05–5 g/mL and *y* = 0.1099*x* + 10.387 with 97.5% accuracy in the concentration range of 10–150 μg/mL. The analysis of these compounds at low concentrations was particularly crucial, since CTX and other anticancer medications had insufficient quantities in human fluids and ambient waters. As seen in Figure 5D, the nonimprinted polymer-coated chips’ surface adsorption was determined to be 8, with the functional groups and CTX forming most hydrogen bonds. On the other hand, molecular imprinting improved the adsorption of CTX on the sensor surfaces, as indicated by the surface adsorption of 27 for the CTX MIP sensor. These analyses determined the imprinting factor (IF) to be Δ*R*(MIP)/Δ*R*(NIP) = 27/8 = 3.4. As a result, the affinity to the CTX molecule was raised by 3.4-folds, indicating that imprinting was effective.

#### Selectivity

Despite their exceptional sensitivity to the target molecule, one of the essential characteristics of the sensors was their extremely low selectivity for other compounds in the environment. Selectivity tests involving the addition of other chemotherapy drugs that are expected to be present in the medium were conducted to assess the sensor’s selectivity.

**Figure 6:**
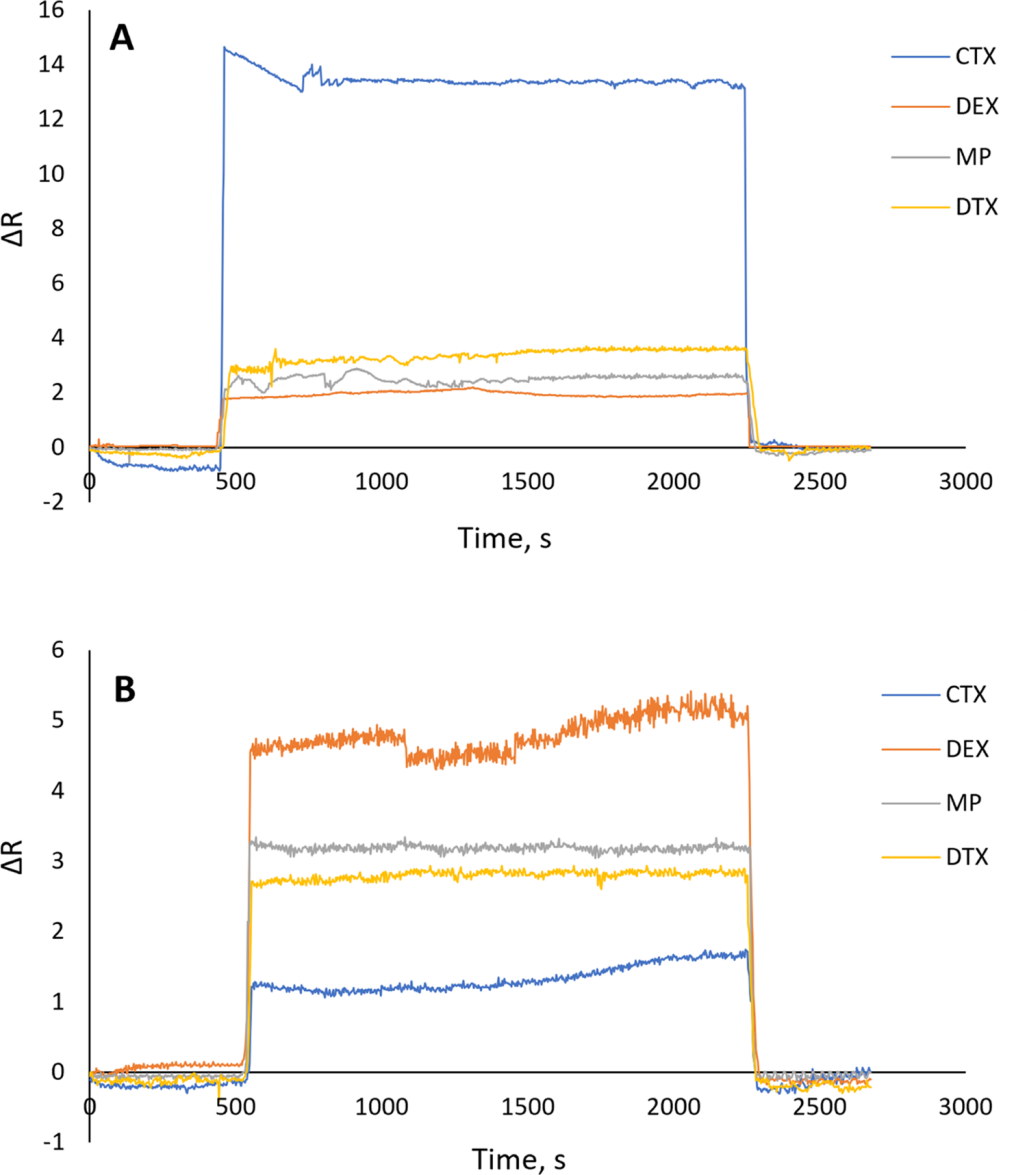
Selectivity study of (A) CTX MIP sensor (*C*_CTX_, *C*_DEX_, *C*_MP_, and *C*_DTX_ equal to 20.0 μg/mL, *T*: 25 °C, pH: 6) and (B) NIP sensor (*C*_CTX_, *C*_DEX_, *C*_MP_, and *C*_DTX_ equal to 20.0 μg/mL, *T*: 25 °C, pH: 6).

We employed DEX, MP, and DTX, three different drugs, for our selectivity investigations. The target molecule’s close structural resemblance determined the selected molecules and their likelihood of existing in the same surroundings. DTX is also a taxoid, antineoplastic agent, and its structural analogue to CTX is used to treat prostate cancer similarly. MP and DTX are administered to reduce the adverse effects of treatment during the treatment period. They reduce allergic reactions and other autoimmune responses.^32^ Because of their usage during the treatment period, they may be in the biological fluid of the patient and analytical matrix, which may obstruct the analysis of CTX, so we choose them for investigation. Each drug was applied to the sensor at 20.0 μg/mL. Figure 6A,B presents the obtained sensorgram, while Table 1 contains the calculated values.

**Table 1:** Selectivity Coefficient of the CTX MIP Sensor

	CTX MIP sensor		NIP sensor		
	Δ*R*	*k*	Δ*R*	*k*	*k*′
CTX	14		1.2		
DEX	1.8	7.8	4.7	0.26	30
MP	2.6	5.4	3.2	0.38	14.2
DTX	3.1	4.5	2.8	0.43	10.5

As shown in Table 1, CTX’s calculated selectivity (eq 1) constants for DTX, MP, and DTX were 7.8, 5.4, and 3.1 (Figure 6A and Table 1), respectively. For nonimprinting sensors, these values were 0.26, 0.38, and 0.43 (Figure 6B and Table 1). The imprinting efficiency of CTX for DTX, MP, and DTX was demonstrated by the computed relative selectivity (eq 2) constants of CTX (30.4, 14.2, and 10.5). As a result, CTX had a better response signal from the imprinting sensor than other drugs.
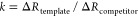
1
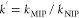
2

#### Adsorption Characteristics and Isotherms

We determined the kinetic parameter using the equation given in the reference.^33^Table 2 contains the calculated parameters. The target molecule’s interaction with the developed sensor and the magnitude of the connection was revealed by the rate constants and equilibrium constants of the adsorption of CTX by the sensor. Table 2 shows that both the association rate constant (0.85 mL/μgs) and the association equilibrium constant (2.02 mL/μg) were more significant than the dissociation rate constant (0.42 1/s) and dissociation equilibrium constant (0.5 μg/mL), respectively. The results demonstrate the high affinity of CTX for the sensor of interest.

**Table 2:** Kinetics Constants for the CTX MIP Sensor

association kinetics analysis		equilibrium analysis (Scatchard)	
*k*_a_(mL/μgs)	0.85	Δ*R*_max_(μg/cm^2^)	23
*k*_d_(1/s)	0.42	*K*_A_(mL/μg)	0.084
*K*_A_(mL/μg)	2.02	*K*_D_(μg/mL)	11.9
*K*_D_(μg/mL)	0.5	*R*^2^	0.91
*R*^2^	0.99		

Langmuir, Freundlich, and Langmuir–Freundlich isotherm models were employed to ascertain the CTX’s adsorption characteristics on the CTX MIP sensor. A detailed explanation of adsorption isotherms is given in our previous article.^34^

Table 3 contains the calculated findings of the adsorption isotherm data. Our data analysis (correlation coefficient and maximum response signal values) revealed that the CTX adsorption feature resembled the Langmuir–Freundlich model. The Langmuir–Freundlich model depicts the behavior of the heterogeneous surface across an extensive concentration range and is appropriate for a system that does not precisely match either system alone.

**Table 3:** CTX MIP Sensor Adsorption Models

Langmuir		Freundlich		Langmuir–Freundlich	
Δ*R*_max_	6.7	Δ*R*_max_	14	Δ*R*_max_	19
*K*_D_	0.06	1/*n*	0.76	1/*n*	0.76
*K*_A_	16.7	*R*^2^	0.978	*K*_D_	0.045
*R*^2^	0.992			*K*_A_	22.2
				*R*^2^	0.998

#### Repeatability and Storability

**Figure 7:**
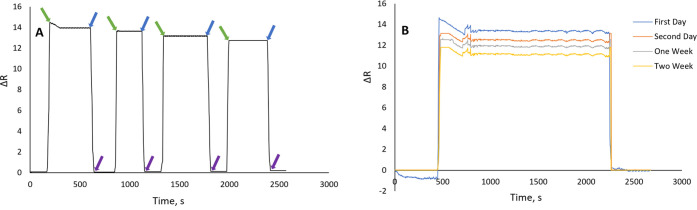
Repeatability and storability study. (A) Repeatability: to ensure consistency, the experiment was carried out three times (*n* = 3) with five different duplicates (green arrow: equilibration; blue arrow: adsorption; purple arrow: regeneration). (B) Storability: CTX MIP sensor sensorgrams obtained on different dates. *(All repeatability and storability studies were repeated three times (*n* = 3), *C*_CTX_: 20.0 μg/L, *t*: 25 °C).

In Figure 7, the repeatability and reusability of the imprinted sensors were examined. For the repeatability experiment, we performed four continuous analytical cycles (repeated three times (*n* = 3)). The CTX concentration was 20.0 μg/mL. The repeatability experiment demonstrated that the CTX MIP sensor retains analysis capacity even after four continuous analysis cycles, performed in Figure 7A.

To test the sensor’s storability, we experimented with Figure 7B on several dates spanning from one day to two weeks. After its manufacture, we chose the first day, second day, first week, and second week (storage condition: pH 6.0 buffer solution, in the refrigerator). The system’s reproducibility studies were determined using precision studies.

For intraday testing (five replicates with three groups), studies on the CTX MIP sensor’s repeatability of the signal response were statistically analyzed, and reproducibility accuracy was confirmed by computing the percent relative standard deviation (% RSD). Expressed as a percent RSD, the results of intraday trials were reported as being less than 1.3, showing strong repeatability. After two weeks, the data indicated that the sensor maintained a required affinity to CTX. Repeatability and storability were examined, and our sensor had excellent repeatability and storability, which was ideal for a sensor because using the same sensor saved money, time, and human labor.

### Confirmation Analysis in Artificial Plasma

The HPLC system confirmed the selective determination of CTX in artificial plasma samples using the CTX MIP sensor. The proposed HPLC method was validated per the International Council for Harmonisation of Technical Requirements for Pharmaceuticals for Human Use (ICH) guidelines. Different concentrations of CTX solutions (0.1–150 μg/mL) were prepared and given to the HPLC system to create a calibration curve. It was decided to compare the results from the SPR method with those from the HPLC method at this point. It was decided to compare the results from the SPR method with those from the HPLC method at this point. The recovery values obtained from the SPR study show parallelism with the data obtained from HPLC. CTX demonstrates linearity over the concentration range from 0.1 to 150 μg/mL (Table 4). *y* = 28,550*x* + 4278.7 (*R*^2^ = 0.9999) was found to be the linear regression equation. The LOD was determined to be 0.0305 μg/mL, whereas the LOQ was determined to be 0.0840 μg/mL. Compared to the HPLC method, the SPR method does not require preliminary preparation, does not require column conditioning, and is easier to implement. An excellent correlation was found between the two analytical techniques (*R*^2^ = 0.9999 for HPLC and *R*^2^ = 0.9974 (0.05–5 μg/mL) and *R*^2^ = 0.9747 (10–150 μg/mL) for SPR). In light of the data obtained, it can be said that the SPR method is reliable.

**Table 4:** Accuracy of the CTX MIP Sensor Method Compared to that of the HPLC Method

	found (μg/mL)		recovery (%)	
prepared concentration (μg/mL)	SPR	HPLC	SPR	HPLC
0.05	0.049	na	97	na
0.1	0.099	0.097	99	97
1	0.098	0.098	98	98
5	4.96	4.97	99	99
10	9.97	9.96	99	99
20	19.96	19.94	99	99
50	49.97	49.95	99	99
100	99.92	99.90	99	99
150	148.6	148.9	99	99

## Conclusions

For the detection of a chemotherapeutic drug, CTX, a molecularly imprinted polymer-based plasmonic SPR sensor, has been effectively synthesized. According to our knowledge, this is the first molecularly imprinted SPR sensor designed for CTX detection. The MAA-EGDMA-HEMA polymer was UV photopolymerized to create the CTX MIP sensor, which was characterized by AFM, ellipsometry, and CA measurements. This method allowed for the rapid and simultaneous analysis of CTX with LOD and LOQ values of 0.012 and 0.036 μg/mL, respectively, at low detection limits. Additionally, the CTX MIP sensor directly measured CTX with excellent accuracy and selectivity. The Langmuir–Freundlich model, which depicts the behavior of the heterogeneous surface across a wide concentration range, was shown to be the most suitable model for the CTX MIP sensor. It was discovered that our sensor had high repeatability and storability, an important feature for any sensor because reusing the same sensor meant less money and time spent developing the sensor. The HPLC system validated the SPR nanofilm sensor’s specific determination of CTX in the artificial plasma sample. The SPR method is simpler to use and requires less previous preparation than the HPLC method, as well as no column conditioning. The two analytical methods showed excellent agreement (*R*^2^ = 0.9999 for HPLC and *R*^2^ = 0.9974 (0.05–5 μg/mL) and 0.9747 (10–150 μg/mL) for SPR). The results allow us to conclude that the SPR approach is reliable.
